# Causal Inference Framework Reveals Mediterranean Diet Superiority and Inflammatory Mediation Pathways in Mortality Prevention: A Comparative Analysis of Nine Common Dietary Patterns

**DOI:** 10.3390/foods14173122

**Published:** 2025-09-06

**Authors:** Jianlin Lin, Qiletian Wang, Xiaoxia Liu, Miao Zhou, Zhongwen Feng, Xiuling Ma, Junrong Li, Renyou Gan, Xu Wang, Kefeng Li

**Affiliations:** 1 Faculty of Applied Sciences, Macao Polytechnic University, Macau 999078, China; p2424411@mpu.edu.mo (J.L.); p2415627@mpu.edu.mo (Q.W.); p2412574@mpu.edu.mo (X.L.); p2412633@mpu.edu.mo (Z.F.); p2412671@mpu.edu.mo (X.M.); p2213983@mpu.edu.mo (J.L.); 2 School of Public Health, Zhengzhou University, Zhengzhou 450001, China; mzhou@gs.zzu.edu.cn; 3 Department of Food Science and Nutrition, The Hong Kong Polytechnic University, Hong Kong 999077, China; renyou.gan@polyu.edu.hk; 4 State Key Laboratory of Food Nutrition and Safety, College of Food Science and Engineering, Tianjin University of Science and Technology, Tianjin 300457, China

**Keywords:** dietary patterns, Mediterranean diet, cardiovascular mortality, all-cause mortality, causal inference, propensity score matching, mediation analysis, inflammatory biomarkers

## Abstract

**Background/Objectives**: While some dietary indices have been developed to assess diet quality and chronic disease risk, their comparative effectiveness within the same population remains unclear due to methodological limitations in observational studies. This study employs a causal inference framework to compare nine dietary indices for reducing all-cause and cardiovascular mortality, while investigating inflammatory pathways through multiple mediation analysis. **Methods**: Using dietary data from 33,881 adults (aged ≥ 20 years, median follow-up 92 months), we applied a causal directed acyclic graph to identify the minimum sufficient adjustment set and implemented generalized propensity score matching to address confounding. Robust Cox proportional hazards regression assessed associations between nine dietary indices—Dietary Inflammatory Index (DII), Composite Dietary Antioxidant Index (CDAI), Healthy Eating Index 2015/2020 (HEI-2015/2020), Alternate Healthy Eating Index (AHEI), Alternate Mediterranean Diet (aMED), Mediterranean Diet Index (MEDI), and Dietary Approaches to Stop Hypertension (DASH/DASHI)—and mortality outcomes. Multiple additive regression trees (MART) algorithm was used for multiple mediation analysis to examine inflammatory markers (PAR, SII, NPR, TyG, LMR, PLR, ELR, CRP) as mechanistic mediators. **Results**: Among 33,881 participants (mean age 47.07 years, 51.34% women), 4,230 deaths occurred, including 827 cardiovascular deaths. Under the causal inference framework, higher DII scores increased both all-cause (HR: 1.07; 95% CI: 1.02–1.12) and cardiovascular mortality risk (HR: 1.07; 95% CI: 1.04–1.10) by 7%. The aMED demonstrated the strongest protective association, reducing all-cause mortality by 12% (HR: 0.88; 95% CI: 0.80–0.97) and cardiovascular mortality by 11% (HR: 0.89; 95% CI: 0.80–0.98), followed by MEDI with similar magnitude effects. Other healthy dietary indices showed modest 1–3% risk reductions. Multiple mediation analysis revealed that inflammatory markers, particularly neutrophil-to-platelet ratio (NPR) and systemic immune-inflammation index (SII), significantly mediated diet–mortality associations across all indices, with C-reactive protein (CRP) serving as the most frequent mediator. **Conclusions**: Using causal inference methodology, the Mediterranean dietary pattern (aMED) shows the strongest causal association with reduced mortality risk, with inflammatory pathways serving as key mediating mechanisms. These findings provide robust evidence for prioritizing Mediterranean dietary patterns in public health interventions and clinical practice, while highlighting inflammation as a critical therapeutic target for dietary interventions aimed at reducing mortality risk.

## 1. Introduction

Cardiovascular disease (CVD) remains a leading global cause of death, with incidence rising from 271 million in 1990 to 523 million in 2020, and mortality increasing from 12.1 million to 18.6 million over the same period [1,2]. Despite substantial advances in pharmacological and interventional treatments, these epidemiological trends continue to impose enormous health and economic burdens worldwide. Diet represents one of the most critical modifiable risk factors for CVD, influencing multiple pathophysiological pathways including systemic inflammation, lipid metabolism, endothelial function, and blood pressure regulation [3,4]. To systematically evaluate diet quality and guide evidence-based dietary interventions, researchers have developed some dietary indices that can be broadly categorized into three types: those based on established dietary guidelines and recommendations (e.g., Healthy Eating Index [HEI], Alternate Healthy Eating Index [AHEI]), those reflecting specific dietary patterns or cultural traditions (e.g., Mediterranean Diet scores), and those derived empirically from statistical clustering of dietary behaviors within populations [5]. Each category offers unique insights into different aspects of dietary quality, yet their development has largely proceeded independently, creating challenges for practitioners and policymakers seeking to identify optimal dietary recommendations.

While these diverse indices all aim to assess diet quality and reduce chronic disease risk, their comparative effectiveness within the same population remains underexplored. Most existing research has focused on individual dietary indices in isolation, limiting our understanding of which patterns may offer superior health benefits. For instance, the Dietary Inflammatory Index (DII) specifically links dietary components to inflammatory biomarkers such as IL-6 and C-reactive protein, emphasizing its mechanistic relevance to CVD-related outcomes [6]. Relying on single indices or limited comparative studies may overlook the nuanced benefits that different dietary approaches might offer across diverse populations and health contexts.

Traditional observational studies examining diet–mortality associations face significant methodological challenges that may lead to biased or inconsistent findings. Conventional approaches often underestimate true dietary effects by inappropriately adjusting for potential mediators (such as chronic diseases) as confounders, thereby blocking causal pathways rather than controlling for confounding [7,8]. Additionally, residual confounding from unmeasured lifestyle factors, socioeconomic variables, and genetic predispositions can substantially bias effect estimates in either direction [9].

Causal inference frameworks offer a principled approach to address these methodological limitations by explicitly defining causal assumptions, identifying appropriate adjustment sets, and employing advanced statistical techniques to minimize bias [10,11,12]. Key components include the use of directed acyclic graphs (DAGs) to visualize causal relationships and identify confounders versus mediators, propensity score methods to balance treatment groups on observed covariates, and sensitivity analyses to assess robustness to unmeasured confounding [13,14]. When properly implemented, these methods can provide more reliable estimates of causal effects that approximate those obtained from randomized controlled trials, which are often infeasible for long-term dietary interventions due to ethical and practical constraints.

Inflammation represents a fundamental mechanism underlying CVD development and progression, with diet serving as a key modulator of inflammatory processes through multiple pathways [15,16]. While extensive research has examined inflammation in the context of the DII, the mediating role of inflammatory markers across other dietary indices remains poorly understood.

Using data from the National Health and Nutrition Examination Survey (NHANES, 2003–2018), this study employs a comprehensive causal inference framework to address critical gaps in our understanding of optimal dietary patterns for mortality reduction. Specifically, we aim to (1) compare the causal associations of nine widely used dietary indices with all-cause and cardiovascular mortality using generalized propensity score matching and robust statistical methods, and (2) investigate the mediating role of inflammatory biomarkers in these relationships through advanced multiple mediation analysis. These findings will provide evidence-based guidance for identifying optimal dietary patterns and inform the development of more effective public health strategies and clinical interventions to reduce mortality risk.

## 2. Materials and Methods

### 2.1. Study Population

This study, which combines cross-sectional NHANES data (2005–2018) with longitudinal mortality follow-up from CDC linkage, enables evaluation of associations between dietary patterns and mortality outcomes. Initially, 70,190 participants (2005–2018) met the inclusion criteria. We applied systematic exclusion criteria to ensure data quality and analytical validity: individuals under 20 years of age (n = 30,440), those with missing dietary quality data (n = 4440), missing mortality linkage information (n = 68), and those with >20% missing covariate data (n = 1360). The final analytical sample comprised 33,881 participants with complete data for causal inference analysis (Figure S1).

### 2.2. Assessment of Dietary Indices

We evaluated nine well-established dietary quality indices representing different theoretical frameworks and dietary patterns: Dietary Inflammatory Index (DII), Composite Dietary Antioxidant Index (CDAI), Alternate Mediterranean Diet Score (aMED), Mediterranean Diet Index based on PREDIMED trial serving sizes (MEDI), Alternate Healthy Eating Index (AHEI), Healthy Eating Index 2015 (HEI-2015), Healthy Eating Index 2020 (HEI-2020), Dietary Approaches to Stop Hypertension Index (DASH), and DASH Index based on trial-specific serving sizes (DASHI).

All dietary indices were calculated using 24 h dietary recall data collected through the NHANES Automated Multiple-Pass Method, which has been validated for accurate nutrient and food group estimation [15,16]. For detailed information on the nine dietary indices, see Supplementary Materials.

### 2.3. Ascertainment of Inflammatory Biomarkers

We included seven inflammatory and metabolic biomarkers as potential mediators based on their established roles in cardiovascular pathophysiology and availability in NHANES: Platelet-to-Lymphocyte Ratio (PLR), Lymphocyte-to-Monocyte Ratio (LMR), Platelet-to-Albumin Ratio (PAR), Neutrophil-to-Platelet Ratio (NPR), Eosinophil-to-Lymphocyte Ratio (ELR), C-reactive protein (CRP), Systemic Immune-Inflammation Index (SII), and Triglyceride–Glucose Index (TyG) [17]. Other inflammatory biomarkers, like IL-6 and TNF-α, exist but were not included due to NHANES data limitations [18].

Laboratory measurements were obtained following standardized NHANES protocols. Lymphocyte, neutrophil, eosinophil, albumin, and platelet counts were measured in cells/μL. Derived indices were calculated as follows: SII = (Platelet count × Neutrophil count)/Lymphocyte count; TyG = ln [Triglycerides (mg/dL) × Plasma Glucose (mg/dL)/2].

### 2.4. Outcome Ascertainment

The primary outcomes were all-cause mortality and cardiovascular disease (CVD) mortality. Mortality status was determined through probabilistic matching with the National Death Index (NDI), maintained by the National Center for Health Statistics, with follow-up through 31 December 2019. The NDI linkage provides high sensitivity (>95%) and specificity (>99%) for mortality ascertainment in NHANES participants

CVD mortality was defined using International Classification of Diseases, 10th Revision (ICD-10) codes: I00–I09 (rheumatic heart diseases), I11 (hypertensive heart disease), I13 (hypertensive heart and kidney disease), and I20–I51 (ischemic heart diseases, pulmonary heart disease, and other forms of heart disease). This definition aligns with standard epidemiological practice and captures the majority of cardiovascular deaths in the U.S. population [19].

### 2.5. Covariate Assessment

Based on established literature and causal assumptions, we identified potential covariates across multiple domains: demographic characteristics [sex (male, female), age (continuous), race/ethnicity (Mexican American, Non-Hispanic Black, Non-Hispanic White, Other Hispanic, Other Race)], socioeconomic factors [education level (<high school, high school, >high school), marital status (married/partnered, single), poverty-income ratio (continuous)], lifestyle behaviors [smoking status (yes, no), alcohol consumption (grams/day, continuous), physical activity (metabolic equivalent minutes/week, quartiles)], and anthropometric measures [BMI (<25, 25–29.9, ≥30 kg/m^2^)].

We also assessed prevalent chronic conditions that could confound diet–mortality associations: diabetes mellitus, hypertension, stroke, coronary heart disease, atherosclerotic cardiovascular disease (ASCVD), and myocardial infarction. These were defined using standard NHANES criteria combining self-reported physician diagnosis, medication use, and relevant laboratory values where applicable.

### 2.6. Causal Inference Framework

*Causal Assumptions and Directed Acyclic Graph*

To estimate causal effects of dietary patterns on mortality outcomes, we implemented a comprehensive causal inference framework based on the potential outcomes model and graphical causal models [20]. We constructed a causal directed acyclic graph (DAG) to explicitly represent our causal assumptions about the relationships between dietary quality, confounders, mediators, and mortality outcomes (Figure S2).

The DAG guided identification of the minimum sufficient adjustment set (MSAS) for unbiased causal effect estimation, which included the following: sex, age, race/ethnicity, education level, marital status, smoking status, alcohol consumption, poverty–income ratio, BMI, and physical activity [21]. Importantly, we did not adjust for chronic diseases (diabetes, hypertension, CVD) as these represent potential mediators rather than confounders in the causal pathway from diet to mortality.

*Missing Data Handling*

To address missing covariate data while preserving the representativeness of our sample, we employed multiple imputation using chained equations (MICE) [22]. We generated 25 imputed datasets using all variables in the analysis model plus auxiliary variables predictive of missingness. The imputation model included interactions and non-linear terms to preserve complex relationships. Results were combined across imputed datasets using Rubin’s rules to obtain valid point estimates and confidence intervals that account for imputation uncertainty.

*Generalized Propensity Score Matching*

For each dietary index (treated as a continuous intervention), we estimated generalized propensity scores using the MSAS identified from the DAG [23]. Unlike binary propensity scores, generalized propensity scores accommodate continuous treatments by modeling the conditional density of the treatment given confounders.

We employed random forests, a flexible machine learning approach, to estimate propensity scores, capturing non-linear relationships and interactions between confounders and dietary quality. The random forest model was implemented with the following parameters: 500 trees, a minimum node size of 5, and a maximum depth of 10, with variable importance assessed using mean decrease in impurity. Hyperparameters were tuned via 10-fold cross-validation to optimize out-of-bag error. Confounders included in the model were age, sex, socioeconomic status, physical activity, and baseline health conditions, selected based on their relevance to dietary quality and outcome as per the DAG. Balance was assessed by examining the distribution of confounders across different levels of dietary quality before and after weighting by inverse propensity scores (Figure S3). Adequate balance was defined as standardized mean differences <0.1 for all confounders.

*Outcome Modeling and Sensitivity Analysis*

After propensity score matching, to address potential data clustering and non-independence, we used robust cox proportional hazards regression to estimate the association between dietary indices and mortality, accounting for the complex survey design through appropriate variance estimation. We tested the proportional hazards assumption using Schoenfeld residuals and employed robust variance estimators to address potential violations. To evaluate the association between dietary quality and health outcomes, we dichotomized each dietary index at the median to define high versus low dietary quality groups. We then estimated the time-varying absolute risk difference (ARD) for mortality between these groups over a 180-month follow-up period, using inverse probability weighting based on generalized propensity scores to adjust for confounders. The ARD represents the absolute difference in cumulative risk between the high and low dietary quality groups at each time point, providing insight into the evolving impact of dietary quality over time.

To assess non-linear dose–response relationships, we fitted restricted cubic splines with knots at the 10th, 50th, and 90th percentiles of each dietary index distribution within the Cox regression framework [24,25]. Non-linearity was tested using likelihood ratio tests comparing spline models to linear models. To evaluate the potential impact of missing value imputation, we conducted a sensitivity analysis by re-performing the analysis of dietary indices and mortality after excluding all missing values. For hazard ratios, E-values were calculated as: when HR > 1, E-Value = $HR+\sqrt{HR*(HR-1)}$; when HR < 1, E-Value = $\frac{1}{HR}+\sqrt{\frac{1}{HR}*\left(\frac{1}{HR}-1\right)}$.

*Multiple Mediation Analysis*

Mediator Selection and Assumptions

We conducted multiple mediation analysis to quantify the extent to which inflammatory biomarkers mediate the relationship between dietary quality and mortality [26]. Potential mediators were selected based on (1) significant association with the dietary exposure (*p* < 0.05), (2) significant association with the outcome when adjusting for the exposure and other mediators (*p* < 0.05), and (3) biological plausibility based on established mechanisms.

The mediation analysis assumes (1) no unmeasured confounding of exposure-outcome, exposure-mediator, and mediator-outcome relationships, (2) no mediator-outcome confounders affected by exposure, and (3) correct model specification. While these assumptions are untestable, we strengthened their plausibility through careful confounder control guided by the DAG.

*Multiple Additive Regression Trees (MART) Algorithm*

We employed the MART algorithm to estimate direct and indirect effects through multiple mediators simultaneously [27]. MART uses machine learning techniques to flexibly model complex, non-linear relationships between exposures, mediators, and outcomes while avoiding parametric assumptions that may be violated in traditional mediation approaches.

The algorithm estimates the following: (1) total effects (exposure → outcome), (2) direct effects (exposure → outcome, not through mediators), and (3) indirect effects (exposure → mediators → outcome). We calculated relative effects (RE) as the ratio of direct or indirect effects to total effects, providing interpretable measures of the proportion of the total effect mediated through inflammatory pathways.

### 2.7. General Statistical Analysis

Due to the complex sampling design of NHANES, we incorporated sample weights to obtain national estimates. Continuous variables are presented as weighted means ± standard errors, or weighted median (M) and interquartile range (IQR). Categorical variables as frequencies and percentages. Differences in continuous and categorical variables between deceased and non-deceased groups were compared using one-way ANOVA, Kruskal–Wallis H test, or chi-square test, as appropriate.

All statistical analyses were performed using R software (4.4.2). Multiple imputations were carried out using the ‘mice’ package (3.18.0), and multiple mediation analyses were conducted using the ‘mma’ package (10.8-1). A two-sided *p*-value < 0.05 was considered statistically significant.

## 3. Results

### 3.1. Sample Population Characteristics

After applying our causal inference framework and exclusion criteria, the final analytical sample comprised 33,881 participants with a mean age of 47.07 years (SE = 0.23) and weighted female representation of 51.34%. During a median follow-up of 92 months, we observed 4230 deaths, including 827 cardiovascular deaths, yielding mortality rates of 12.5% and 2.4%, respectively.

Baseline characteristics differed significantly between survivors and decedents, supporting the need for rigorous confounder adjustment (Table 1). Participants who died from cardiovascular disease or any cause were predominantly male (54.12% and 52.88% vs. 48.32%), older (mean ages 70.18 and 66.89 vs. 45.46 years), less educated, more likely to be unmarried, and had higher smoking prevalence (all *p* < 0.001). Chronic disease burden was substantially higher among decedents, with cardiovascular mortality cases showing particularly elevated prevalence of diabetes (46.97% vs. 20.09%), hypertension (76.50% vs. 34.54%), and established cardiovascular disease (40.93% vs. 6.46%).

Among dietary indices, significant baseline differences between survivors and decedents were observed for DII (higher inflammatory potential among decedents: 1.68 vs. 1.37, *p* < 0.001), HEI-2015, HEI-2020, DASH, CDAI, and MEDI scores. Inflammatory biomarkers showed consistent patterns, with decedents exhibiting higher pro-inflammatory profiles including elevated PLR, reduced LMR, lower PAR (indicating hypoalbuminemia), and increased TyG index (all *p* < 0.001).

Correlation analysis revealed important relationships among dietary indices (Figure S4). DII and CDAI showed strong negative correlation (r = −0.83), confirming their inverse relationship regarding inflammatory potential. Mediterranean diet variants (aMED, MEDI) demonstrated moderate positive correlations with guideline-based indices (AHEI, HEI-2015, HEI-2020; r > 0.5), suggesting shared dietary quality components while maintaining distinct scoring emphases.

### 3.2. Causal Effects of Dietary Quality on Mortality Outcomes

**Figure 1:**
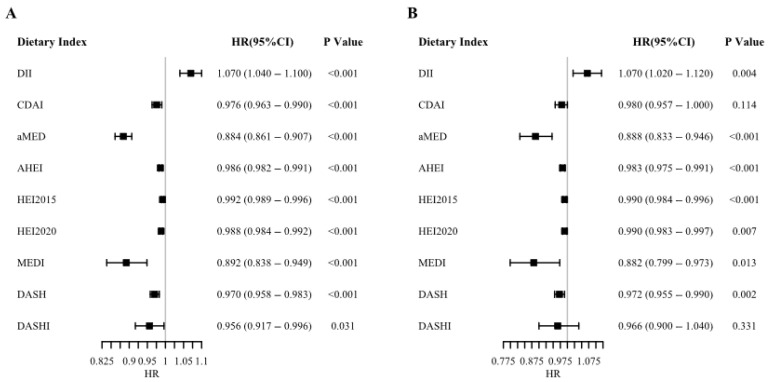
Uncovering Dietary Impact: Associations of Nine Dietary Indices with Cardiovascular and All-Cause Mortality. Panel (**A**) reveals the associations between nine dietary indices and all-cause mortality, while Panel (**B**) highlights their links to cardiovascular mortality. Adjusted for age, sex, education, poverty level, smoking status, alcohol consumption, and exercise, this analysis underscores the varying protective or detrimental roles of diet quality in mortality risk. Abbreviations: DII = Dietary Inflammatory Index; MED = Mediterranean Diet; HEI = Healthy Eating Index; AHEI = Alternative Healthy Eating Index; DASH = Dietary Approaches to Stop Hypertension; DASHI = Dietary Approaches to Stop Hypertension Index.

Using our causal inference framework with generalized propensity score matching, we estimated the causal effects of nine dietary indices on mortality outcomes (Figure 1). After adjusting for the minimum sufficient adjustment set identified through directed acyclic graph analysis, dietary inflammatory index (DII) showed consistent harmful associations, increasing both cardiovascular mortality (HR 1.07, 95% CI 1.04–1.10) and all-cause mortality (HR 1.07, 95% CI 1.02–1.12) by 7% per unit increase.

In contrast, healthy dietary patterns demonstrated potential protective causal effects, with the alternate Mediterranean Diet (aMED) showing the strongest associations. The aMED reduced all-cause mortality by 12% (HR 0.88, 95% CI 0.80-0.97) and cardiovascular mortality by 11% (HR 0.89, 95% CI 0.80–0.98) per unit increase. The PREDIMED-based Mediterranean Diet Index (MEDI) showed similarly strong protective effects (11% reduction for both outcomes). Other healthy dietary indices showed more modest but statistically significant protective associations: CDAI reduced mortality risk by approximately 3% (HR 0.97, 95% CI 0.96–0.99), while AHEI, HEI-2015, and HEI-2020 each conferred 1–2% risk reductions per unit increase.

Notably, the DASH Index (DASHI) showed no significant association with cardiovascular mortality when treated as a continuous variable, despite DASH showing modest protective effects. This finding persisted in quartile-based analyses (Supplemental Results and Figure S5), suggesting that adherence to DASH principles may require threshold effects or longer observation periods to manifest cardiovascular benefits in this population.

To elucidate the potential clinical significance of dietary indices, we calculated the absolute risk difference (ARD). The results demonstrated that, for both all-cause mortality and cardiovascular mortality, the ARD for all dietary indices increased steadily over time, starting near 0 at baseline and diverging progressively to approximately 3% by 150 months (Figure S6). Overall, these findings highlight that higher dietary quality, as measured by various indices, is associated with lower absolute risks over time, with the magnitude of this effect varying by index. Notably, for all-cause mortality, the MED dietary index exhibited the strongest protective effect, with an ARD of 3.6%.

### 3.3. Dose–Response Relationships Through Restricted Cubic Splines

**Figure 2:**
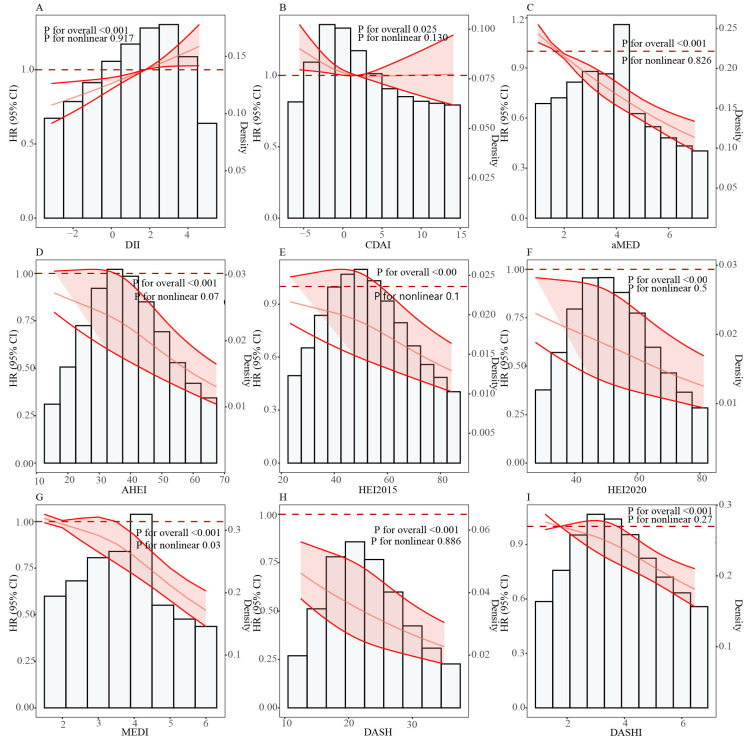
Illuminating Dose–Response Dynamics: Dietary Quality and All-Cause Mortality Risk. This figure elucidates the dose–response relationships between dietary indices and all-cause mortality, with panels (**A**–**I**) examining DII, CDAI, aMED, MEDI, AHEI, HEI-2015, HEI-2020, DASH, and DASHI, respectively. Panels (**A**–**I**) assess associations between distinct dietary indices and mortality risk. Adjusted for age, sex, education, poverty level, smoking status, alcohol consumption, and exercise, these analyses highlight linear trends critical for tailoring dietary interventions. In all subfigures, the red line represents the RCS curve with its confidence interval. Abbreviations: DII = Dietary Inflammatory Index; MED = Mediterranean Diet; HEI = Healthy Eating Index; AHEI = Alternative Healthy Eating Index; DASH = Dietary Approaches to Stop Hypertension; DASHI = Dietary Approaches to Stop Hypertension Index.

Restricted cubic spline analysis within the Cox regression framework revealed predominantly linear dose–response relationships between dietary quality and mortality outcomes (Figure 2). For DII, we observed a monotonic increase in all-cause mortality risk with higher inflammatory dietary scores (*p* for non-linearity >0.05), confirming that even modest increases in dietary inflammatory potential translate to measurably higher mortality risk.

Conversely, healthy dietary indices demonstrated linear protective dose–response relationships. The aMED showed the steepest protective slope, with mortality risk declining consistently across the entire score distribution. Similar linear patterns were observed for MEDI, AHEI, HEI-2015, HEI-2020, DASH, and CDAI (all *p* for trend <0.05). The absence of threshold effects suggests that incremental improvements in dietary quality provide proportional mortality benefits, supporting population-wide dietary improvement strategies rather than targeting only high-risk individuals. A similar pattern was observed in the association between diet and cardiovascular mortality (Supplemental Results, Figure S7).

### 3.4. Inflammatory Mediation Pathways

Correlation analysis revealed significant relationships among inflammatory biomarkers (Figure S8). SII and PLR exhibited the strongest positive association (r = 0.66), while LMR showed the strongest negative association, with NPR displaying a moderate association (r = 0.34). Among other pairs, CRP and SII demonstrated the most robust positive correlation (r = 0.27), followed by TyG (r = 0.24) and NPR (r = 0.18). Additionally, LMR exhibited negative correlations with the majority of inflammatory markers.

**Figure 3:**
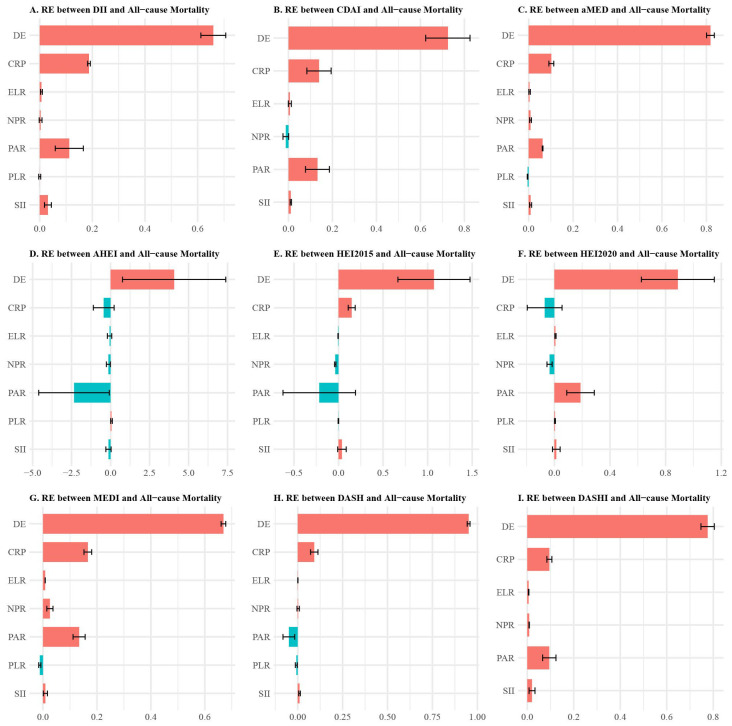
Decoding Inflammation’s Role: Multiple Mediation Analysis of Inflammatory Markers in Diet and All-Cause Mortality. This figure unveils the relative effects of inflammatory markers (e.g., PAR, SII, NPR) mediating the association between dietary quality and all-cause mortality, with panels (**A**–**I**) examining DII, CDAI, aMED, MEDI, AHEI, HEI-2015, HEI-2020, DASH, and DASHI, respectively. Panels (**A**–**I**) assess mediations between distinct dietary indices and mortality risk. Adjusted for age, sex, education, poverty level, smoking status, alcohol consumption, and exercise, these findings illuminate inflammation’s pivotal role in diet-related mortality risk. In all subfigures, green represents a negative effect (protective association with reduced mortality risk), while red indicates a positive effect (association with increased mortality risk). Abbreviations: DII = Dietary Inflammatory Index; MED = Mediterranean Diet; HEI = Healthy Eating Index; AHEI = Alternative Healthy Eating Index; DASH = Dietary Approaches to Stop Hypertension; DASHI = Dietary Approaches to Stop Hypertension Index; PLR = Platelet-to-Lymphocyte Ratio; LMR = Lymphocyte-to-Monocyte Ratio; PAR = Platelet-to-Albumin Ratio; SII = Systemic Inflammation Index; NPR = Neutrophil-to-Platelet Ratio; ELR = Eosinophil-to-Lymphocyte Ratio; CRP = C-reactive Protein; TyG = Triglyceride–Glucose Index.

Multiple mediation analysis using the MART algorithm revealed complex inflammatory pathways mediating diet-mortality associations (Figure 3). For all-cause mortality, C-reactive Protein (CRP), neutrophil-to-platelet ratio (NPR) and systemic immune-inflammation index (SII) emerged as the most consistent mediators across dietary indices, participating in mediation pathways for all nine indices examined.

The inflammatory mediation patterns varied meaningfully across dietary indices. For DII, the strongest positive mediators were CRP, platelet-to-albumin ratio (PAR), SII, and NPR, with eosinophil-to-lymphocyte ratio (ELR) serving as a negative mediator. This pattern suggests that pro-inflammatory diets increase mortality risk primarily through enhanced systemic inflammation and reduced nutritional status (reflected by low albumin).

Mediterranean dietary patterns (aMED, MEDI) showed distinctive mediation profiles, with CRP, PAR, SII, NPR as primary positive mediators. Remarkably, the association between HEI2015 and all-cause mortality was predominantly mediated by CRP, whereas in HEI2020, PAR primarily mediated its association with all-cause mortality.

For cardiovascular mortality, similar mediation patterns were observed (Figure S9), with CRP, PAR, NPR, SII, and lymphocyte-to-monocyte ratio (LMR) serving as the most frequent mediators. The consistent involvement of TyG in cardiovascular mortality mediation highlights the importance of metabolic-inflammatory pathways in cardiovascular disease progression. The involvement of LMR suggests that Mediterranean diets may influence mortality through modulation of adaptive immune responses in addition to innate inflammatory pathways.

### 3.5. Sensitivity Analysis for Unmeasured Confounding

To evaluate the potential risks associated with missing value imputation, we conducted a sensitivity analysis by excluding all missing values. The results were consistent with our primary findings, indicating that the MED dietary pattern remains the potentially optimal dietary practice for preventing all-cause mortality and cardiovascular mortality, with risk reductions exceeding 10% (Figure S10).

**Figure 4:**
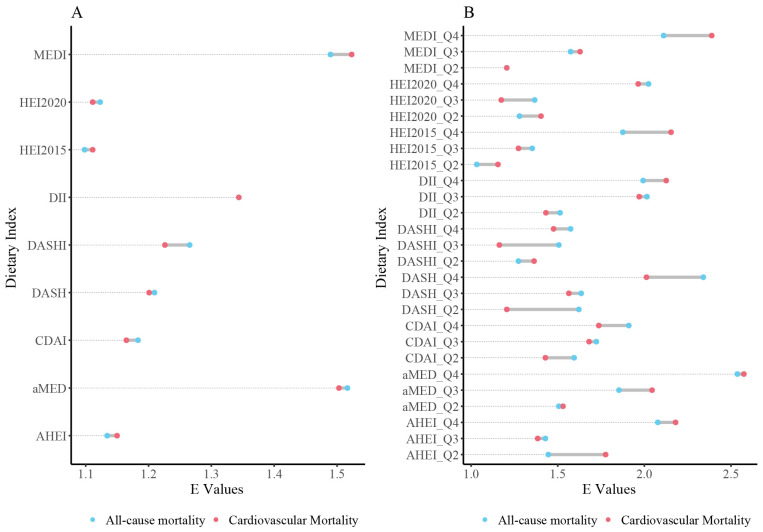
Strengthening Causal Insights: E-Values for Dietary Quality and Mortality Associations. (**A**) quantifies E-values for continuous dietary indices and their associations with cardiovascular and all-cause mortality, while Panel (**B**) extends this to quartile-based indices. These analyses highlight the robustness of findings against unmeasured confounding, reinforcing the causal link between diet and mortality outcomes. Abbreviations: DII = Dietary Inflammatory Index; MED = Mediterranean Diet; HEI = Healthy Eating Index; AHEI = Alternative Healthy Eating Index; DASH = Dietary Approaches to Stop Hypertension; DASHI = Dietary Approaches to Stop Hypertension Index.

Furthermore, E-value analysis demonstrated substantial robustness of our causal estimates to potential unmeasured confounding (Figure 4). For all-cause mortality associations with continuous dietary indices, E-values ranged from 1.10 to 1.53, while quartile-based analyses yielded E-values from 1.09 to 2.53. Similarly, cardiovascular mortality E-values ranged from 1.09 to 1.53 (continuous) and 1.09 to 2.52 (categorical).

These E-values indicate that unmeasured confounders would need to be associated with both dietary quality and mortality with effect sizes comparable to or exceeding those of smoking (HR ≈ 1.6–2.0) to fully explain away our observed associations. Given our comprehensive adjustment for known strong confounders including smoking, socioeconomic factors, and lifestyle behaviors, such strong unmeasured confounding appears unlikely.

The highest E-values were observed for Mediterranean dietary patterns (aMED, MEDI), reflecting both their stronger effect sizes and greater robustness to confounding. Even moderate unmeasured confounders (HR ≈ 1.3–1.4) would be insufficient to nullify these associations, providing strong evidence for causal relationships between Mediterranean dietary adherence and reduced mortality risk.

## 4. Discussion

### 4.1. Principal Findings and Causal Evidence

This study represents the first comprehensive application of causal inference methodology to compare nine dietary indices for mortality reduction within the same population. Using directed acyclic graphs to identify confounders, generalized propensity score matching to address confounding, and multiple mediation analysis to elucidate mechanisms, we provide evidence consistent with causal inference that dietary patterns substantially influence mortality risk through inflammatory pathways.

Our causal analysis revealed that the alternate Mediterranean Diet (aMED) conferred the strongest protective effects, reducing all-cause mortality by 12% and cardiovascular mortality by 11% per unit increase. The Dietary Inflammatory Index (DII) demonstrated consistent harmful effects, increasing both mortality outcomes by 7% per unit increase. Other healthy dietary indices showed modest but meaningful protective effects (1–3% risk reductions). Importantly, restricted cubic spline analysis revealed predominantly linear dose–response relationships, indicating that incremental dietary improvements provide proportional mortality benefits without threshold effects.

The application of causal inference methodology addresses critical limitations of previous observational studies that may have underestimated dietary effects through inappropriate confounder adjustment. Our E-value analysis demonstrates that unmeasured confounders would need effect sizes comparable to smoking (HR ≈ 1.6–2.0) to fully explain away our findings, providing strong evidence for causal relationships rather than spurious associations.

### 4.2. Mechanistic Insights Through Multiple Mediation Analysis

A key innovation of this study was the systematic investigation of inflammatory mediation pathways across all dietary indices using the MART algorithm. Our findings reveal that neutrophil-to-platelet ratio (NPR) and systemic immune-inflammation index (SII) serve as universal mediators, participating in diet-mortality pathways across all nine indices examined. This consistency suggests that anti-inflammatory mechanisms represent a common pathway through which diverse dietary patterns influence mortality risk [19,28,29,30].

These mechanistic insights have important clinical implications. The identification of specific inflammatory biomarkers as key mediators suggests potential targets for monitoring dietary intervention effectiveness and identifying individuals most likely to benefit from specific dietary approaches. Furthermore, the universal involvement of CRP, NPR and SII across dietary patterns suggests these biomarkers could serve as integrative measures of dietary quality and inflammation-related health risk [31]. Clinically, NPR and SII are reliable markers of systemic inflammation in conditions like cardiovascular and metabolic disorders, with elevated levels linked to worse outcomes. Their routine use in blood tests supports clinical monitoring and personalized dietary interventions. Correlation analysis showed moderate positive associations with CRP (CRP-SII: r = 0.27; CRP-NPR: r = 0.18), suggesting NPR and SII complement CRP by capturing broader inflammatory responses. Combining these biomarkers could improve inflammation assessment related to dietary patterns.

### 4.3. Mediterranean Diet Superiority and Mechanistic Basis

The superior performance of Mediterranean dietary patterns aligns with extensive epidemiological evidence and randomized controlled trials demonstrating cardiovascular benefits [32,33]. However, our causal inference approach provides new insights into why Mediterranean diets outperform other healthy dietary patterns. The mediation analysis suggests that Mediterranean diets uniquely modulate multiple inflammatory pathways simultaneously, including systemic inflammation (SII), immune cell ratios (LMR), and nutritional-inflammatory status (PAR).

The Mediterranean dietary pattern’s emphasis on anti-inflammatory foods—including omega-3 rich fish, polyphenol-containing olive oil and wine, antioxidant-rich fruits and vegetables, and fiber-rich legumes—provides a mechanistic basis for its superior anti-inflammatory effects. Unlike other dietary indices that focus on single aspects (e.g., sodium reduction in DASH, antioxidant capacity in CDAI), the Mediterranean pattern provides a comprehensive anti-inflammatory nutritional profile that addresses multiple pathways simultaneously.

However, the regional specificity and cultural adaptability of Mediterranean dietary patterns present implementation challenges in diverse populations. Our findings support the Dietary Guidelines for Americans’ recommendation to choose dietary patterns based on individual health status, preferences, and cultural background while emphasizing anti-inflammatory principles that can be adapted across different food traditions.

### 4.4. DASH Diet Findings and Clinical Implications

The modest effects observed for DASH and DASHI patterns warrant careful interpretation within the causal inference framework. When treated as continuous variables, DASH showed limited cardiovascular mortality benefits, and DASHI showed no significant association. This finding aligns with recent target trial emulation studies using the Alpha Omega cohort, which found no significant mortality reduction with DASH adherence in post-myocardial infarction patients [34].

Several factors may explain these findings within our causal framework. First, medication effects may attenuate DASH benefits, as participants with cardiovascular disease commonly use antihypertensive and lipid-lowering therapies that address similar pathways targeted by DASH [34]. Specifically, medications such as statins and ACE inhibitors target lipid profiles and blood pressure, respectively, which are key mechanisms through which DASH exerts its effects. This overlap may mask the independent contribution of DASH, particularly in populations with high medication adherence, reducing the observed effect size in observational studies. Second, the surrogate endpoint paradox suggests that while DASH effectively reduces blood pressure and weight in short-term trials, these intermediate outcomes may not translate directly to mortality benefits in observational settings [35]. This paradox may be exacerbated by treatment effects, as medications may already optimize these intermediate endpoints, leaving limited room for dietary interventions to demonstrate additional benefits in mortality outcomes. Third, the mixed population in NHANES may dilute associations that might be stronger in hypertensive individuals specifically. For DASHI, the weaker associations could also stem from its broader dietary focus, which may not specifically target cardiovascular pathways as effectively as DASH, especially in medicated populations where treatment effects dominate.

Importantly, our E-value analysis indicates that even for DASH’s modest associations, strong unmeasured confounders (stronger than smoking, HR = 1.63) would be required to nullify the observed effects (E-value = 2.56), supporting the plausibility of causal relationships despite smaller effect sizes [36]. However, the potential for residual confounding by treatment intensity (e.g., dosage or combination therapies) or unmeasured lifestyle factors (e.g., physical activity) should be considered, as these could further explain the weaker-than-expected associations for both DASH and DASHI.

### 4.5. Strengths and Limitations

Although our study is based on a causal inference framework and, assuming the assumptions of causal inference are met, our conclusions somewhat address questions typically answered by RCTs, our study still has limitations. First, the dietary indices were measured using 24 h recall reports, making it difficult to account for dietary changes or assess the duration of dietary adherence. The reliance on 24 h dietary recalls introduces potential recall bias, as participants may inaccurately report their food intake due to memory lapses or social desirability, which could misclassify dietary patterns and attenuate observed associations with mortality outcomes. Additionally, a single 24 h recall may not capture habitual dietary patterns, as day-to-day variations in food intake could misrepresent long-term adherence to diets like DASH or DASHI. Second, dietary indices and disease were measured simultaneously, making it challenging to rule out the impact of disease on diet. For instance, cardiovascular disease patients may be more inclined to follow a high DASH score diet. This temporal alignment issue complicates causal inference, as the cross-sectional nature of the dietary assessment precludes establishing whether dietary patterns preceded disease onset or were adopted in response to a diagnosis, potentially introducing reverse causation bias. Furthermore, the lack of longitudinal dietary data limits our ability to assess how dietary changes over time align with the extended follow-up period for mortality outcomes, which may further dilute the observed associations. Third, we did not explore the association between specific dietary elements and the risk of cardiovascular mortality and all-cause mortality, considering the synergistic effects of dietary elements. Lastly, the generalizability of our findings may be limited, as the included population may not be representative of Asian populations.

## 5. Conclusions

This study suggests that Mediterranean dietary patterns provide superior greater protection consistent with causal inference against mortality (11–12% risk reduction) compared to other healthy dietary indices, operating primarily through anti-inflammatory mechanisms mediated by neutrophil-to-platelet ratio and systemic immune-inflammation index. Using a rigorous causal inference framework with directed acyclic graphs and generalized propensity score matching, we demonstrate that inflammatory pathways universally mediate di-et-mortality associations across all dietary patterns examined. These findings support prioritizing Mediterranean dietary principles in clinical practice and public health pol-icy, while incorporating inflammatory biomarkers as monitoring tools for dietary intervention effectiveness. The linear dose–response relationships observed support population-wide dietary improvement strategies, and the methodological approach demonstrates that causal inference methods can provide robust evidence for dietary interventions within observational data constraints, establishing a framework for future nutrition research that approximates randomized trial evidence.

## Figures and Tables

**Table 1:** Survey-Weighted Characteristics of the NHANES Sample CVD and All-Cause Mortality.

	Total	Alive	CVD Mortality	*p* Value	All-Causal Mortality	*p* Value
**Age, mean (SE)**	47.07 (0.23)	45.46 (0.22)	70.18 (0.49)	**<0.0001**	66.89 (0.35)	**<0.0001**
**Sex, N (%)**				**0.004**		**<0.0001**
Female	17,271 (51.34)	15,791 (51.68)	434 (45.88)		1480 (47.12)	
Male	16,610 (48.66)	14,580 (48.32)	621 (54.12)		2030 (52.88)	
**Race, N (%)**				**<0.0001**		**<0.0001**
Mexican American	5326 (8.36)	5038 (8.74)	72 (3.37)		288 (3.65)	
Non-Hispanic Black	7253 (11.00)	6512 (11.00)	228 (11.84)		741 (11.04)	
Non-Hispanic White	14,601 (67.98)	12,437 (67.05)	660 (78.68)		2164 (79.38)	
Other Hispanic	3183 (5.40)	3003 (5.66)	60 (2.82)		180 (2.17)	
Other Race	3518 (7.27)	3381 (7.56)	35 (3.29)		137 (3.75)	
**Education, N (%)**				**<0.0001**		**<0.0001**
<High school	3488 (5.24)	2894 (5.40)	185 (13.70)		594 (12.38)	
High school	8555 (20.96)	7216 (22.50)	399 (38.63)		1339 (38.55)	
>High school	18,185 (61.99)	16,815 (72.10)	406 (47.66)		1370 (49.07)	
**Marital status, N (%)**				**<0.0001**		**<0.0001**
Married	20,315 (63.85)	18,575 (64.86)	505 (49.86)		1740 (51.73)	
Single	13,556 (36.12)	11,787 (35.14)	550 (50.14)		1769 (48.27)	
**Smoke, N (%)**				**<0.0001**		**<0.0001**
No	18,635 (55.04)	17,276 (56.42)	470 (45.33)		1359 (38.18)	
Yes	15,236 (44.94)	13,086 (43.58)	585 (54.67)		2150 (61.82)	
**Diabetes, N (%)**				**<0.0001**		**<0.0001**
No	24,368 (77.15)	22,514 (79.91)	524 (53.03)		1854 (56.55)	
Yes	8871 (21.58)	7223 (20.09)	530 (46.97)		1648 (43.45)	
**Hypertension, N (%)**				**<0.0001**		**<0.0001**
No	19,739 (62.83)	18,758 (65.46)	237 (23.50)		981 (30.49)	
Yes	14,140 (37.17)	11,612 (34.54)	818 (76.50)		2528 (69.51)	
**CVD, N (%)**				**<0.0001**		**<0.0001**
No	30,286 (91.63)	27,973 (93.54)	591 (59.07)		2313 (68.22)	
Yes	3591 (8.36)	2394 (6.46)	464 (40.93)		1197 (31.78)	
**Stroke, N (%)**				**<0.0001**		**<0.0001**
No	32,567 (97.11)	29,503 (97.90)	890 (86.46)		3064 (88.61)	
Yes	1273 (2.79)	838 (2.10)	160 (13.54)		435 (11.39)	
**ASCVD, N (%)**				**<0.0001**		**<0.0001**
No	30,611 (92.35)	28,164 (94.02)	638 (63.06)		2447 (71.97)	
Yes	3265 (7.64)	2202 (5.98)	417 (36.94)		1063 (28.03)	
**Heart attack, N (%)**				**<0.0001**		**<0.0001**
No	32,402 (96.60)	29,422 (97.55)	839 (81.40)		2980 (86.25)	
Yes	1432 (3.30)	912 (0.45)	213 (18.60)		520 (13.75)	
**DII, mean (SE)**	1.39 (0.03)	1.37 (0.03)	1.65 (0.07)	**<0.0001**	1.68 (0.04)	**<0.0001**
**HEI2015, mean (SE)**	50.71 (0.19)	50.63 (0.19)	52.20 (0.53)	**0.004**	51.71 (0.35)	**0.002**
**DASH, mean (SE)**	2.38 (0.02)	2.37 (0.02)	2.54 (0.07)	**0.01**	2.49 (0.04)	**0.001**
**CDAI, mean (SE)**	0.84 (0.04)	0.90 (0.04)	0.06 (0.13)	**<0.0001**	0.08 (0.08)	**<0.0001**
**MED, mean (SE)**	3.50 (0.02)	3.50 (0.02)	3.56 (0.06)	0.22	3.47 (0.04)	0.48
**AHEI, mean (SE)**	39.08 (0.19)	39.12 (0.20)	39.05 (0.49)	0.24	38.66 (0.32)	0.14
**HEI2020, mean (SE)**	51.46 (0.18)	51.40 (0.18)	53.08 (0.52)	**0.004**	52.17 (0.33)	**0.02**
**MEDI, mean (SE)**	3.59 (0.02)	3.59 (0.02)	3.49 (0.05)	**<0.001**	3.48 (0.03)	**<0.001**
**DASHI, mean (SE)**	3.52 (0.01)	3.52 (0.01)	3.62 (0.06)	0.16	3.55 (0.03)	0.24
**PLR, M (IQR)**	118.57 (94.50, 148.57)	118.18 (94.41, 147.50)	130.00 (98.67, 178.00)	**<0.0001**	125.29 (95.93, 168.62)	**<0.0001**
**LMR, M (IQR)**	3.80 (3.00, 4.80)	3.83 (3.00, 4.83)	3.00 (2.20, 4.00)	**<0.0001**	3.17 (2.33, 4.20)	**<0.0001**
**PAR, M (IQR)**	33.62 (16.51, 65.71)	34.81 (17.50, 67.30)	16.64 (5.30, 36.17)	**<0.0001**	18.49 (6.09, 42.45)	**<0.0001**
**NPR, M (IQR)**	0.02 (0.01, 0.02)	0.02 (0.01, 0.02)	0.02 (0.01, 0.02)	**<0.0001**	0.02 (0.01, 0.02)	**<0.0001**
**ELR, M (IQR)**	0.08 (0.05, 0.13)	0.08 (0.05, 0.13)	0.11 (0.06, 0.17)	**<0.0001**	0.10 (0.06, 0.15)	**<0.0001**
**CRP, M (IQR)**	0.18 (0.07, 0.43)	0.17 (0.07, 0.41)	0.26 (0.11, 0.60)	<0.0001	0.24 (0.10, 0.57)	**<0.0001**
**TyG, mean (SE)**	8.60 (0.01)	8.58 (0.01)	8.84 (0.03)	**<0.0001**	8.81 (0.02)	**<0.0001**

Abbreviations: DII = Dietary Inflammatory Index; MED = Mediterranean Diet; HEI = Healthy Eating Index; AHEI = Alternative Healthy Eating Index; DASH = Dietary Approaches to Stop Hypertension; DASHI = Dietary Approaches to Stop Hypertension Index; PLR = Platelet-to-Lymphocyte Ratio; LMR = Lymphocyte-to-Monocyte Ratio; PAR = Platelet-to-Albumin Ratio; NPR = Neutrophil-to-Platelet Ratio; ELR = Eosinophil-to-Lymphocyte Ratio; CRP = C-reactive Protein; TyG = Triglyceride–Glucose Index.

## Data Availability

The data in this study is available at https://wwwn.cdc.gov/nchs/nhanes/Default.aspx (accessed on 10 August 2025).

## Supplementary Materials

The following supporting information can be downloaded at https://www.mdpi.com/article/10.3390/foods14173122/s1. Figure S1: Study design and flowchart of the selection of eligible study population. Figure S2: Directed Acyclic Graph of Diet Scores and Mortality. Figure S3: Assessment of Dataset Balance After Propensity Score Matching. Figure S4. Correlation Matrix of Nine Diet Scores. Figure S5: Association between dietary quality and cardiovascular and all-cause mortality. Figure S6: Dose–response relationship between dietary quality and cardiovascular mortality. Figure S7: Dose–response relationship between dietary quality and cardiovascular mortality. A. Dose–response relationship between DII and cardiovascular mortality; B. Dose–response relationship between CDAI and cardiovascular mortality; C. Dose–response relationship between aMED and cardiovascular mortality; D. Dose–response relationship between MEDI and cardiovascular mortality; E. Dose–response relationship between AHEI and cardiovascular mortality; F. Dose–response relationship between HEI-2015 and cardiovascular mortality; G. Dose–response relationship between HEI-2020 and cardiovascular mortality; H. Dose–response relationship between DASH and cardiovascular mortality; I. Dose–response relationship between DASHI and cardiovascular mortality. Figure S8: Correlation Matrix of Eight Inflammatory Biomarkers. Figure S9: Relative Effects of Inflammatory Markers that Explain association between diet dietary quality and cardiovascular mortality. Figure S10: Uncovering Dietary Impact: Associations of Nine Dietary Indices with Cardiovascular and All-Cause Mortality after excluding missing value. Panel A reveals the associations between nine dietary indices and all-cause mortality, while Panel B highlights their links to cardiovascular mortality. Adjusted for age, sex, education, poverty level, smoking status, alcohol consumption, and exercise, this analysis underscores the varying protective or detrimental roles of diet quality in mortality risk.

## Abbreviations

The following abbreviations are used in this manuscript:

DGA	Dietary Guidelines for Americans
RCT	Randomized Controlled Trial
DII	Dietary Inflammatory Index
MED	Mediterranean Diet
HEI	Healthy Eating Index
AHEI	Alternative Healthy Eating Index
DASH	Dietary Approaches to Stop Hypertension
DASHI	Dietary Approaches to Stop Hypertension Index
PLR	Platelet-to-Lymphocyte Ratio
LMR	Lymphocyte-to-Monocyte Ratio
PAR	Platelet-to-Albumin Ratio
SII	Systemic Inflammation Index
NPR	Neutrophil-to-Platelet Ratio
ELR	Eosinophil-to-Lymphocyte Ratio
TyG	Triglyceride-Glucose Index
HR	Hazard Ratio
CI	Confidence Interval
NHANES	National Health and Nutrition Examination Survey
CRP	C-reactive Protein
