# Supplementary material for: Causal Inference Framework Reveals Mediterranean Diet Superiority and Inflammatory Mediation Pathways in Mortality Prevention: A Comparative Analysis of Nine Common Dietary Patterns
Source: Foods. 2025 Sep 6;14(17):3122. doi: 10.3390/foods14173122 (PMC12427978; doi:10.3390/foods14173122)
Supplement: Supplementary file 1 [file foods-14-03122-s001.zip › foods-3840587-supplementary.pdf]

Causal Inference Framework Reveals Mediterranean Diet Su-periority and Inflammatory Mediation Pathways in Mortality Prevention: A Comparative Analysis of Nine Common Dietary Patterns

Methods and Materials

Assessment of Dietary Indices

Dietary Inflammatory Index (DII): Measures the impact of diet on inflammation, ranging from -8.87 to 7.98, with higher scores indicating stronger anti-inflammatory effects.

CDAI: Evaluates six dietary components (zinc, selenium, carotenoids, and vitamins A, C, and E) by calculating sex-specific standardized scores for each component and summing them.

MED: Assesses adherence to the Mediterranean diet, with scores ranging from 0 to 9.

MEDI: Similar to MED, this index also evaluates adherence to the Mediterranean diet but includes additional components such as extra virgin olive oil or nuts, with scores ranging from 0 to 11.

AHEI: The Alternative Healthy Eating Index measures the intake of vegetables, fruits, whole grains, nuts, protein sources (e.g., fish and legumes), saturated fats, sugar, and dietary diversity, with scores ranging from 0 to 100.

HEI-2015: Evaluates intake of vegetables, fruits, whole grains, protein sources, healthy fats, saturated fats, sugar, and sodium, with scores ranging from 0 to 100.

HEI-2020: The latest version of the HEI, based on current dietary guidelines and nutritional research, with scores ranging from 0 to 100.

DASH: Assesses dietary intake of vegetables, fruits, whole grains, low-fat dairy products, and low sodium, with scores ranging from 8 to 40.

DASHI: Based on the DASH diet, this index evaluates nine nutritional indicators (fat, saturated fat, protein, cholesterol, fiber, magnesium, calcium, sodium, and potassium), with scores ranging from 0 to 9.

Statistical Analysis

Stable Unit Treatment Value Assumption (SUTVA): The potential outcomes for any individual's dietary quality are unaffected by the dietary quality assigned to other individuals. Additionally, for each individual, different forms or versions of dietary quality do not lead to different potential outcomes.

Consistency: The observed outcome under a specific dietary quality for each individual equals the potential outcome under that dietary quality.

Ignorability: Given the covariates, different levels of dietary quality are independent of mortality (cardiovascular mortality).

Positivity: For any covariate value, the probability of each participant receiving different levels of dietary quality is positive.

RESULTS

Association Between Dietary Quality and Cardiovascular and All-Cause Mortality

To further investigate the association, dietary scores were divided into quartiles, with the first quartile (Q1) serving as the reference. For all-cause mortality, Q2, Q3, and Q4 of DII increased the risk by 13% (95% CI 1.00-1.28), 34% (95% CI 1.00-1.28), and 33% (95% CI 1.00-1.28), respectively. In contrast, Q2, Q3, and Q4 of CDAI decreased the risk by 14% (95% CI 0.83-0.90), 18% (95% CI 0.77-0.88), and 23% (95% CI 0.72-0.83), respectively. MED in Q2, Q3, and Q4 reduced the risk by 11% (95% CI 0.81-0.98), 21% (95% CI 0.74-0.85), and 23% (95% CI 0.63-0.64), respectively. Q4 of AHEI, Q4 of HEI-2020, and Q4 of DASHI reduced the risk by 18% (95% CI 0.61-0.86), 26% (95% CI 0.65-0.85), and 13% (95% CI 0.84-0.90), respectively. HEI-2015 in Q3 and Q4 reduced the risk by 17% (95% CI 0.91-0.95) and 22% (95% CI 0.74-0.82), respectively. MEDI in Q2, Q3, and Q4 reduced the risk by 9% (95% CI 0.88-0.93), 9% (95% CI 0.87-0.95), and 27% (95% CI 0.68-0.79), respectively. Q4 of DASH and DASHI reduced the risk by 33% (95% CI 0.57-0.79) and 13% (95% CI 0.84-0.90), respectively (Supplemental

Figure S4).

For cardiovascular mortality, Q3 and Q4 of DII increased the risk by 32% (95% CI 1.11-1.57) and 39% (95% CI 1.11-1.72), respectively. MED in Q2, Q3, and Q4 reduced the risk by 12% (95% CI 0.80-0.98), 16% (95% CI 0.60-0.91), and 28% (95% CI 0.60-0.65), respectively. Q4 of AHEI, Q4 of HEI-2015, Q4 of HEI-2020, Q4 of MEDI, and Q4 of DASHI reduced the risk by 34% (95% CI 0.55-0.80), 29% (95% CI 0.58-0.88), 24% (95% CI 0.64-0.90), 29% (95% CI 0.61-0.82), and 10% (95% CI 0.86-0.94), respectively (Supplemental Figure S5).

Role of Inflammatory Markers in the Association Between Dietary Quality and Mortality

For cardiovascular mortality, the positive mediator for DII was SII and CRP, and LMR, while the negative mediator was TyG. For CDAI, the top three positive mediators were NPR, CRP, and TyG. For MED, the positive mediators were CRP. SII and NPR, and the negative mediator was LMR. For AHEI, the negative mediators were NPR, LMR and TyG. HEI-2015 showed no statistically significant direct effect; its positive mediator was PAR, and the negative mediator was CRP. For HEI-2020,the positive mediators werePAR, and the negative mediator was SII. MEDI showed no statistically significant direct effect. For DASH, the positive mediator was PAR and CRP. For DASHI, the top three positive mediators were CRP, SII, and LMR. For cardiovascular mortality, CRP, PAR, NPR, SII, and TyG were the most frequent mediators, mediating the association between all dietary indices and cardiovascular mortality (Supplemental Figure S9).

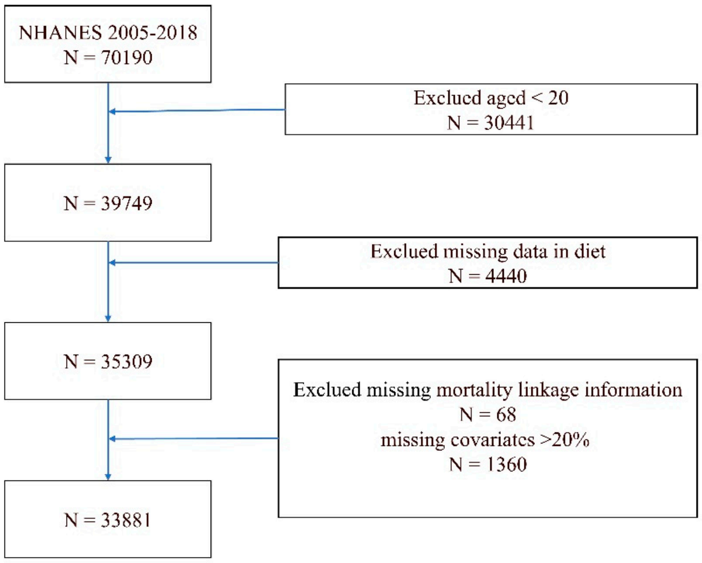

Figure S1. Study design and flowchart of the selection of eligible study population.

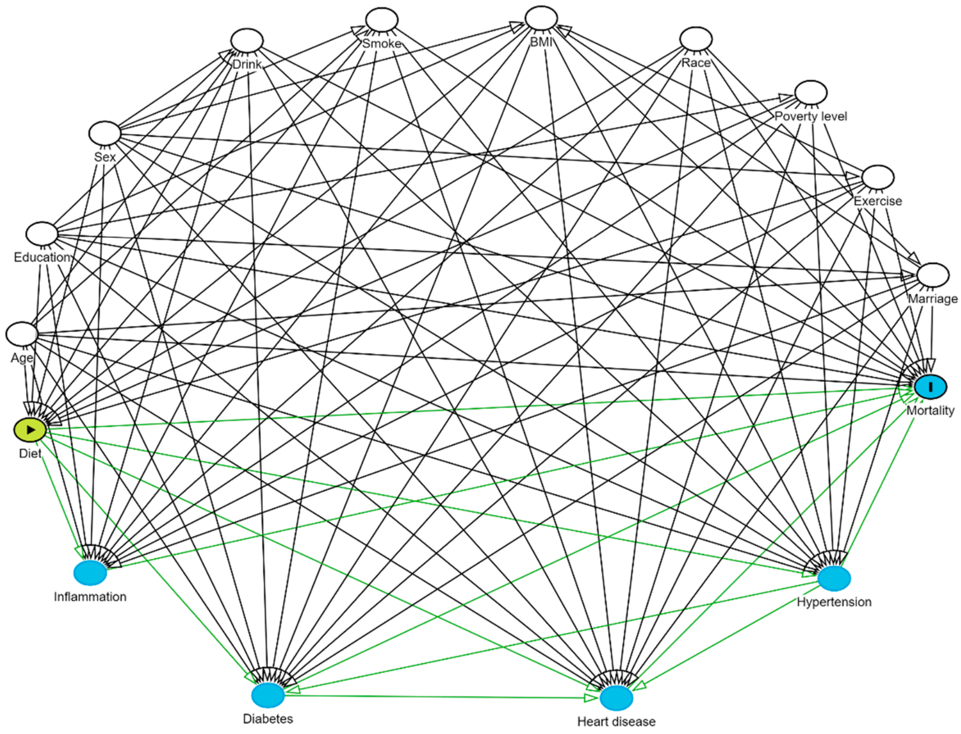

Figure S2. Directed Acyclic Graph of Diet Scores and Mortality.

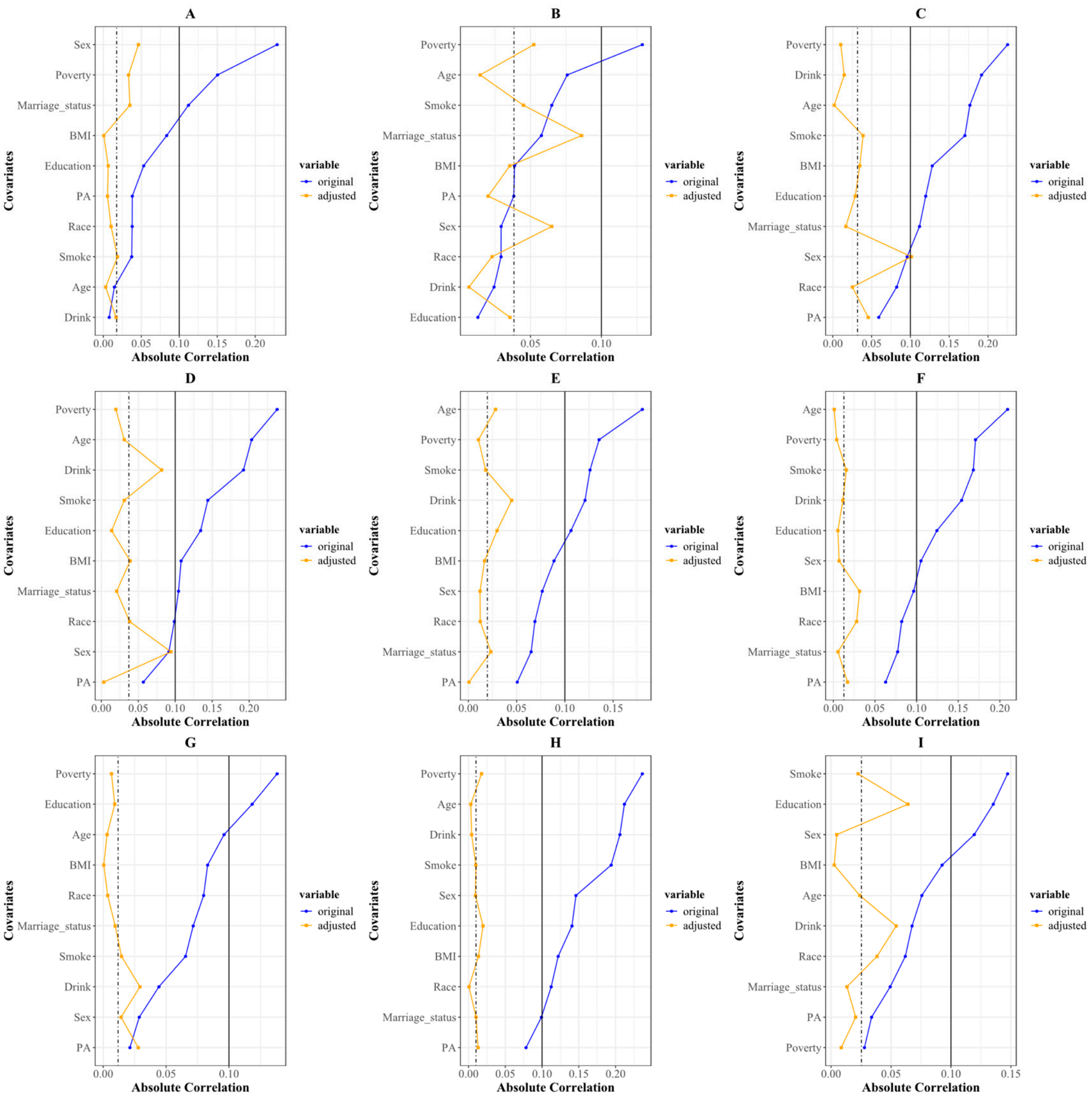

Figure S3. Assessment of Dataset Balance After Propensity Score Matching. A. PSM dataset for DII; B. PSM dataset for CDAI; C. PSM dataset for aMED; D. PSM dataset for MEDI; E. PSM dataset for AHEI; F. PSM dataset for HEI-2015; G. PSM dataset for HEI-2020; H. PSM dataset for DASH; I. PSM dataset for DASHI. Abbreviations: DII = Dietary Inflammatory Index; MED = Mediterranean Diet; HEI = Healthy Eating Index; AHEI = Alternative Healthy Eating Index; DASH = Dietary Approaches to Stop Hypertension; DASHI = Dietary Approaches to Stop Hypertension Index

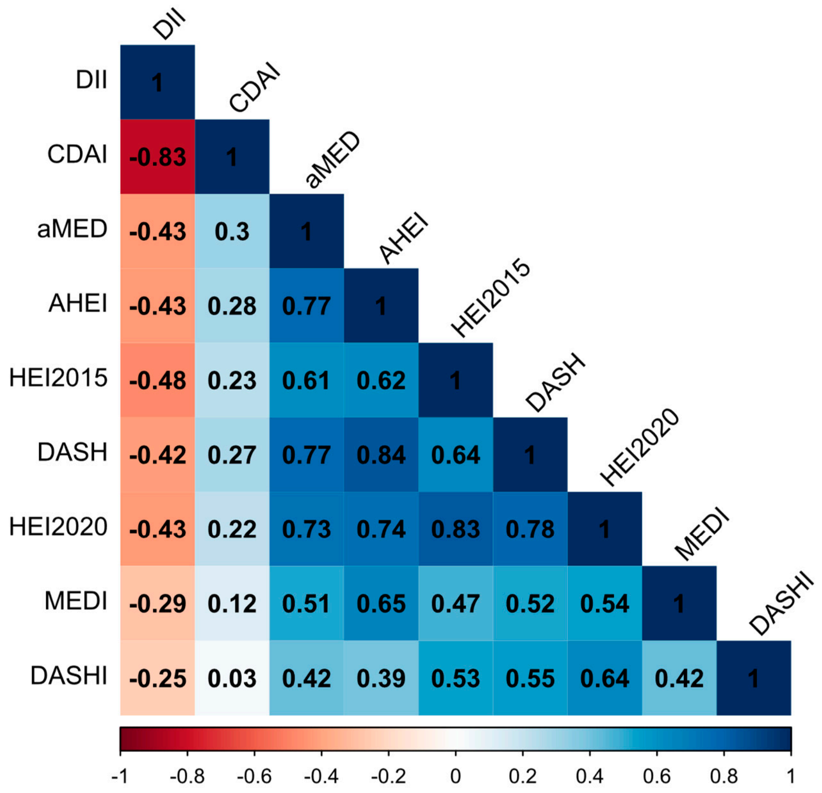

Figure S4. Correlation Matrix of Nine Diet Scores.

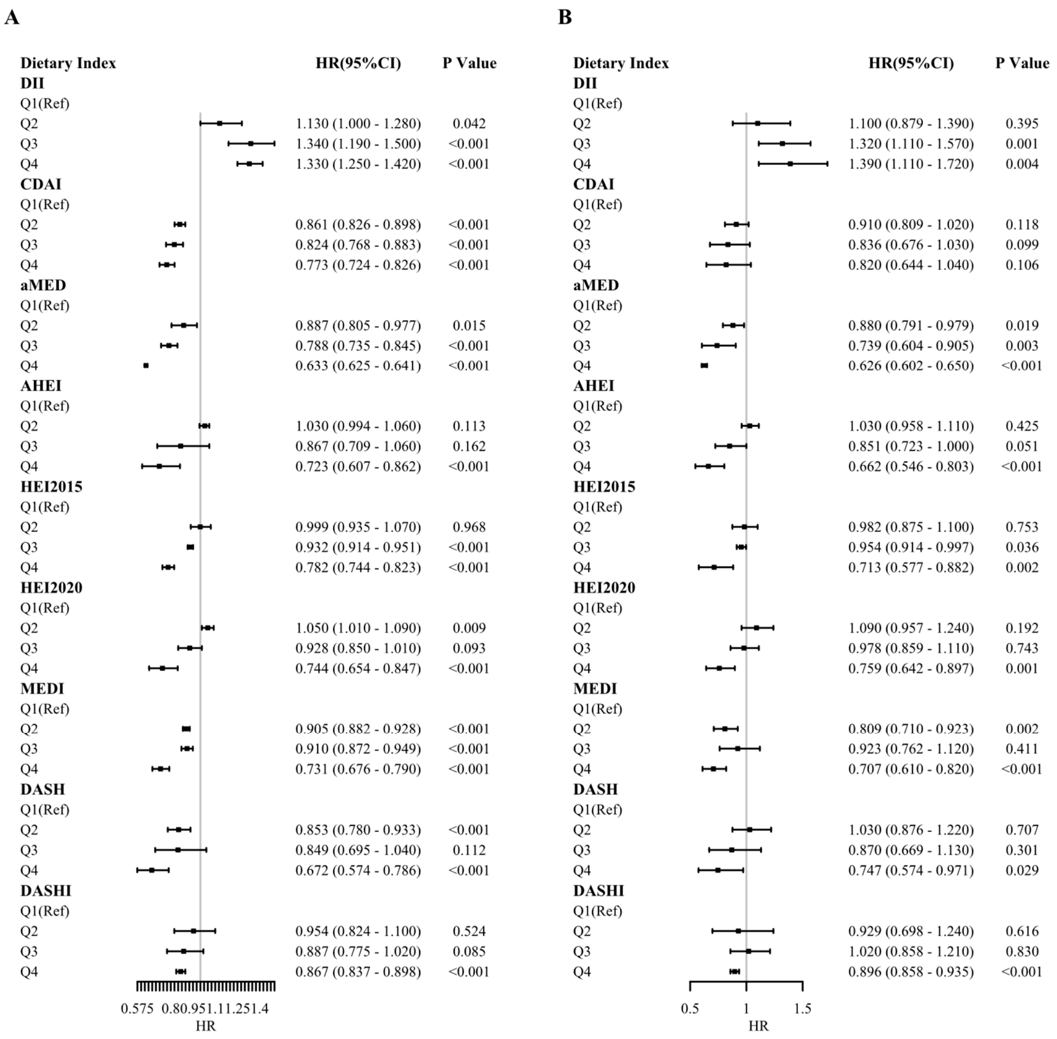

Figure S5. Association between dietary quality and cardiovascular and all-cause mortality. A. Association between dietary quality and all-cause mortality. B. Association between dietary quality and cardiovascular mortality. Abbreviations: DII = Dietary Inflammatory Index; MED = Mediterranean Diet; HEI = Healthy Eating Index; AHEI = Alternative Healthy Eating Index; DASH = Dietary Approaches to Stop Hypertension; DASHI = Dietary Approaches to Stop Hypertension Index.

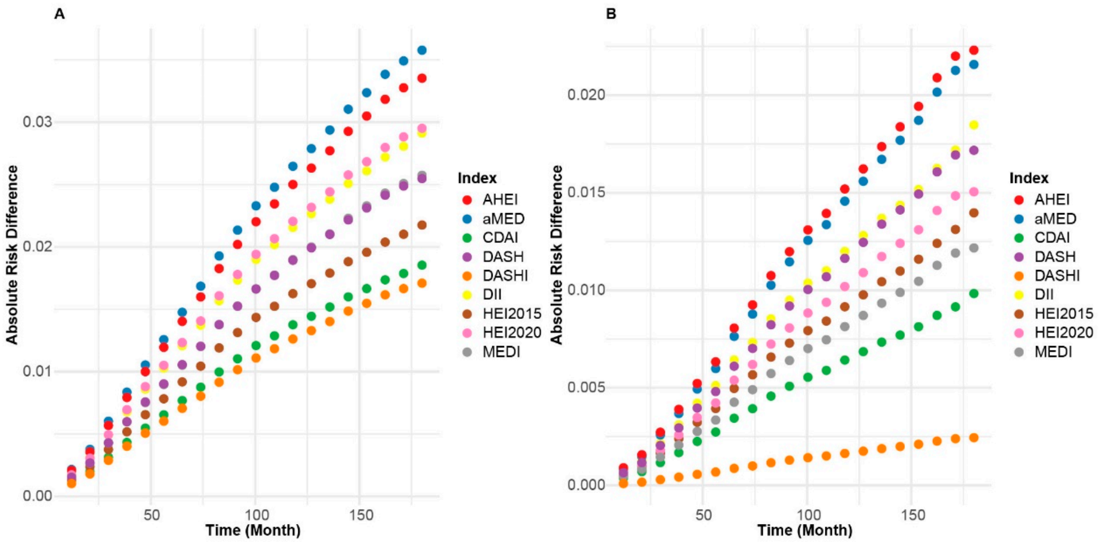

Figure S6. Absolute risk differences between dietary quality and cardiovascular and all-cause mortality. A. Association between dietary quality and all-cause mortality. B. Association between dietary quality and cardiovascular mortality. Abbreviations: DII = Dietary Inflammatory Index; MED = Mediterranean Diet; HEI = Healthy Eating Index; AHEI = Alternative Healthy Eating Index; DASH = Dietary Approaches to Stop Hypertension; DASHI = Dietary Approaches to Stop Hypertension Index.

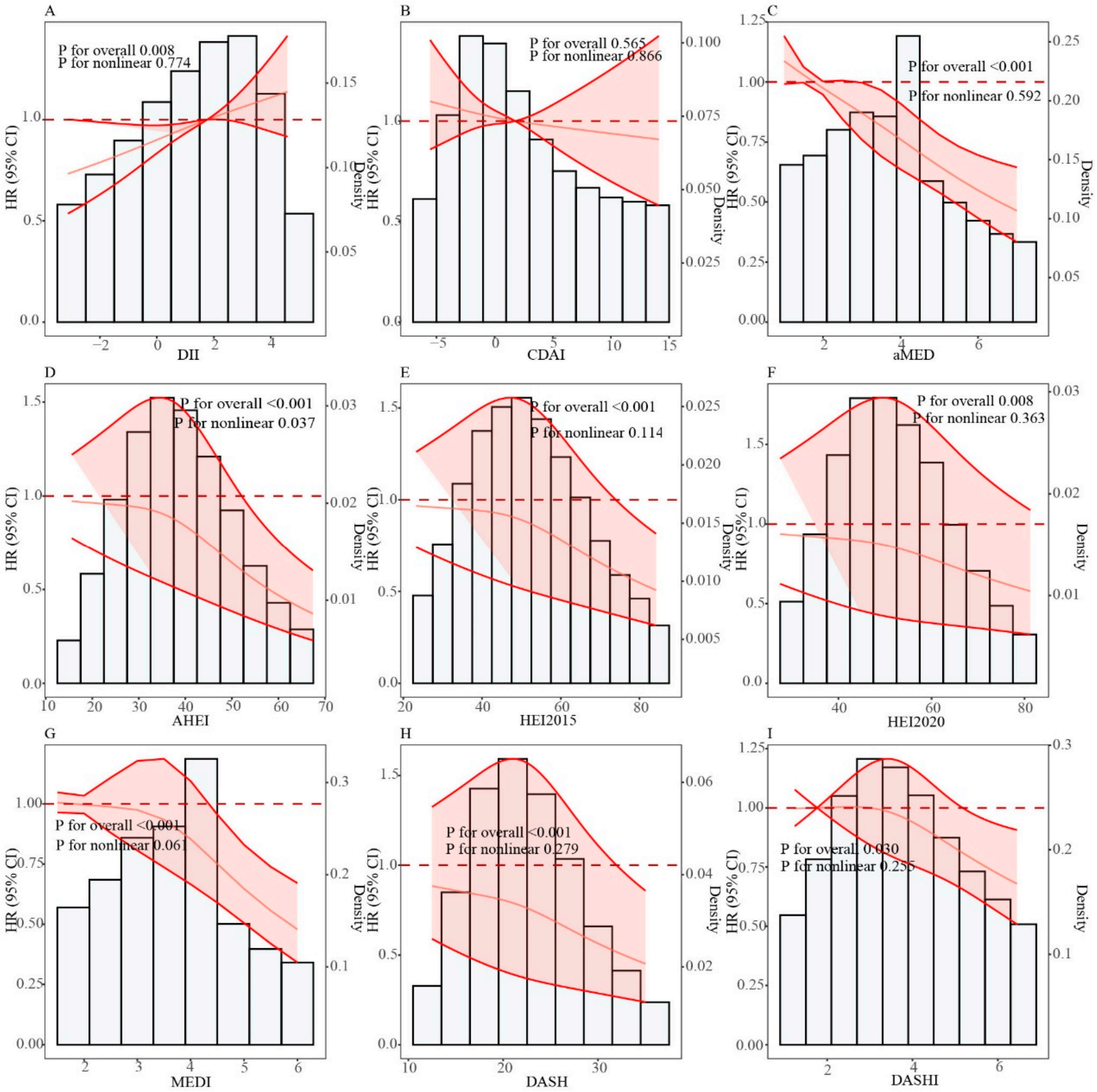

Figure S7. Dose–response relationship between dietary quality and cardiovascular mortality. A. Dose–response relationship between DII and cardiovascular mortality; B. Dose–response relationship between CDAI and cardiovascular mortality; C. Dose–response relationship between aMED and cardiovascular mortality; D. Dose–response relationship between MEDI and cardiovascular mortality; E. Dose–response relationship between AHEI and cardiovascular mortality; F. Dose–response relationship between HEI-2015 and cardiovascular mortality; G. Dose–response relationship between HEI-2020 and cardiovascular mortality; H. Dose–response relationship between DASH and cardiovascular mortality; I. Dose–response relationship between DASHI and cardiovascular mortality. Abbreviations: DII = Dietary Inflammatory Index; MED = Mediterranean Diet; HEI = Healthy Eating Index; AHEI = Alternative Healthy Eating Index; DASH = Dietary Approaches to Stop Hypertension; DASHI = Dietary Approaches to Stop Hypertension Index

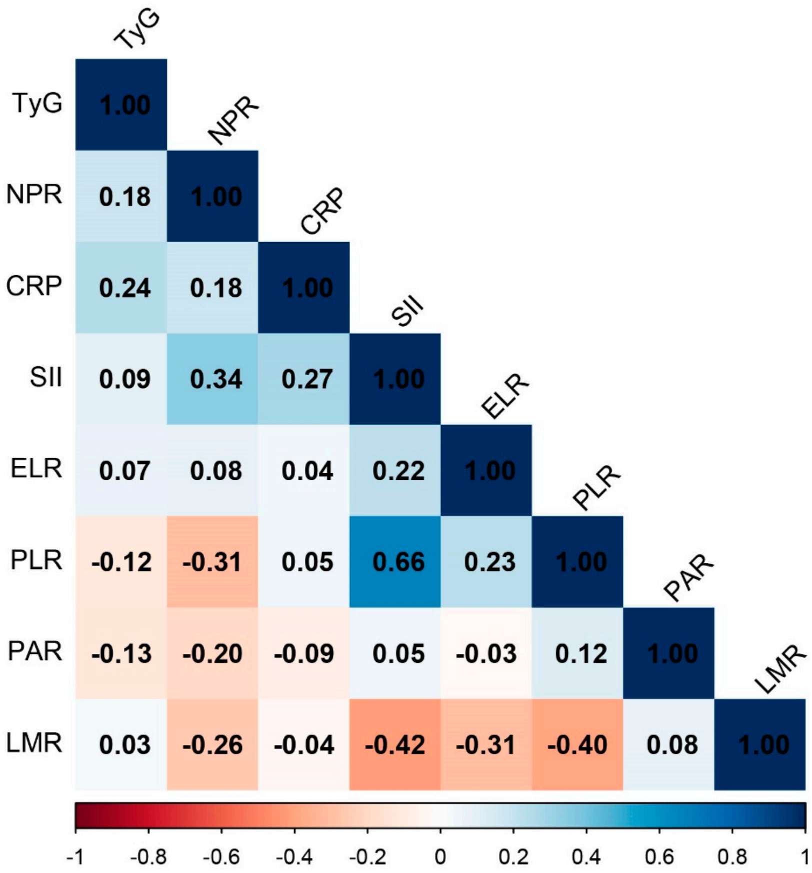

Figure S8. Correlation Matrix of Eight Inflammatory Biomarkers. PLR = Platelet-to-Lymphocyte Ratio; LMR = Lymphocyte-to-Monocyte Ratio; PA = Platelet-to-Albumin Ratio; SII = Systemic Inflammation Index; NPR = Neutrophil-to-Platelet Ratio; ELR = Eosinophil-to-Lymphocyte Ratio; CRP = C-reactive Protein; TyG = Triglyceride-Glucose Index

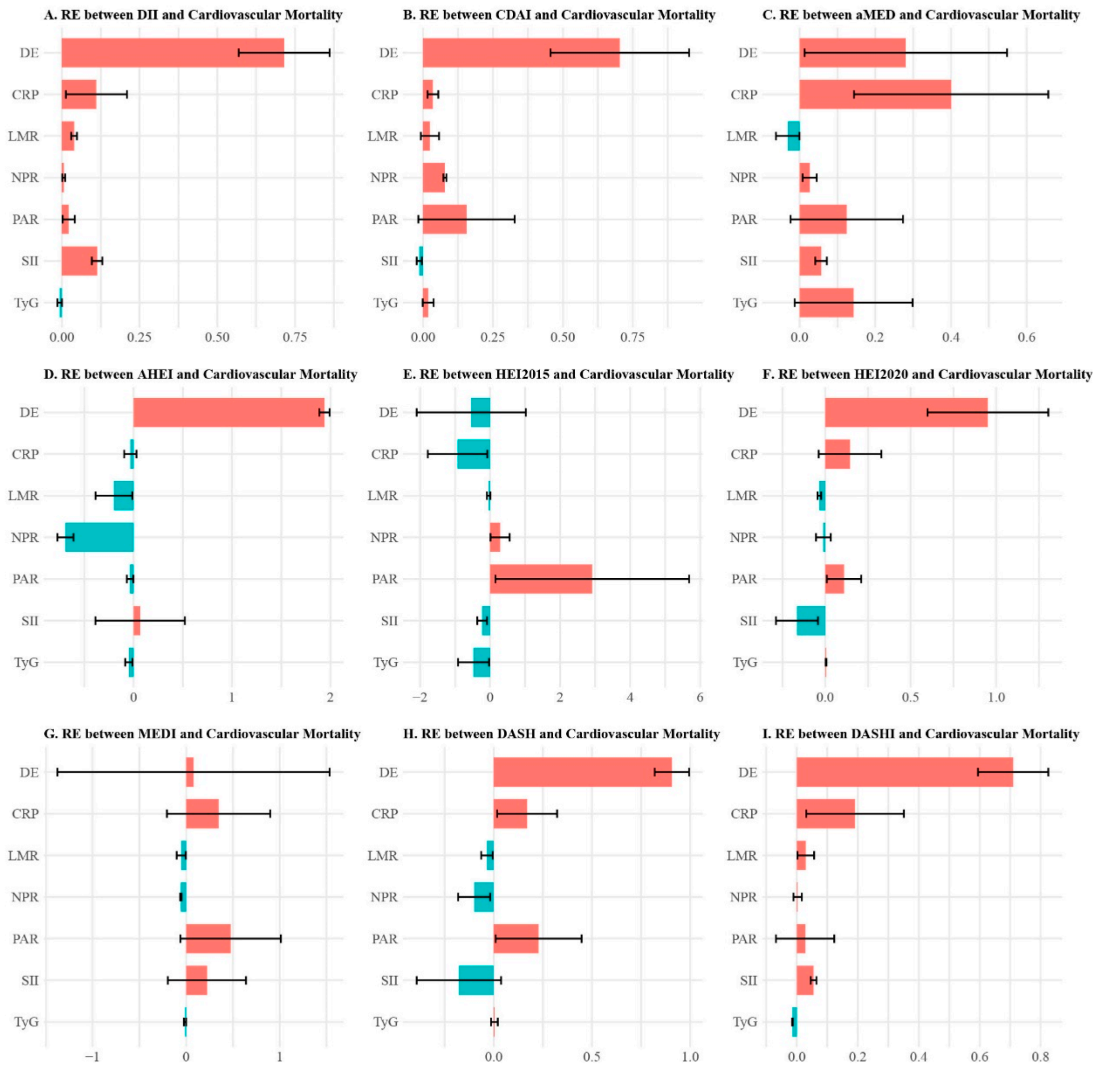

Supplemental Figure S9. Relative Effects of Inflammatory Markers that Explain association between diet dietary quality and cardiovascular mortality. Abbreviations: DII = Dietary Inflammatory Index; MED = Mediterranean Diet; HEI = Healthy Eating Index; AHEI = Alternative Healthy Eating Index; DASH = Dietary Approaches to Stop Hypertension; DASHI = Dietary Approaches to Stop Hypertension Index; PLR = Platelet-to-Lymphocyte Ratio; LMR = Lymphocyte-to-Monocyte Ratio; PA = Platelet-to-Albumin Ratio; SII = Systemic Inflammation Index; NPR = Neutrophil-to-Platelet Ratio; ELR = Eosinophil-to-Lymphocyte Ratio; CRP = C-reactive Protein; TyG = Triglyceride-Glucose Index

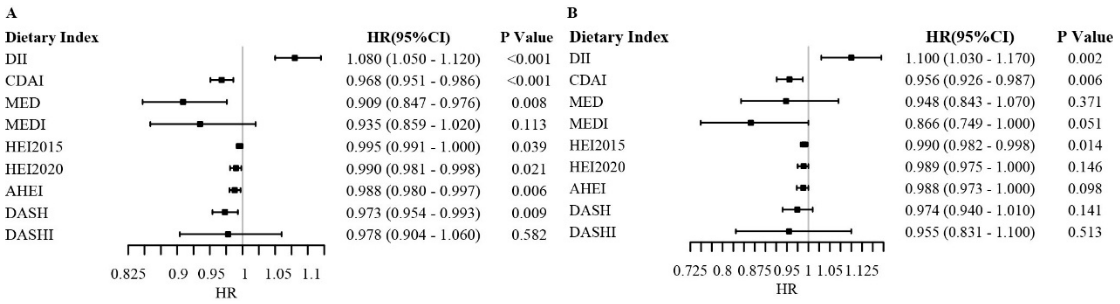

Supplemental Figure S10. Uncovering Dietary Impact: Associations of Nine Dietary Indices with Cardiovascular and All-Cause Mortality after excluding missing value. Panel A reveals the associations between nine dietary indices and all-cause mortality, while Panel B highlights their links to cardiovascular mortality. Adjusted for age, sex, education, poverty level, smoking status, alcohol consumption, and exercise, this anal-ysis underscores the varying protective or detrimental roles of diet quality in mortality risk. Ab-breviations: DII = Dietary Inflammatory Index; MED = Mediterranean Diet; HEI = Healthy Eating Index; AHEI = Alternative Healthy Eating Index; DASH = Dietary Approaches to Stop Hyper-tension; DASHI = Dietary Approaches to Stop Hypertension Index.
